# A Height Nonlinear Velocity Field Algorithm for CORS Station Based on GARCH Model

**DOI:** 10.3390/s22197589

**Published:** 2022-10-06

**Authors:** Hengjing Zhang, Huanling Liu, Dongdong Cui, Fang Zhang

**Affiliations:** 1 School of Geomatics, Liaoning Technical University, Fuxin 123000, China; 2 Chinese Academy of Surveying & Mapping, Beijing 100036, China; 3 Liaoning Electric Power Survey & Design Institute Co., Ltd., China Energy Engineering Group, Shenyang 110179, China

**Keywords:** CORS height time series, nonlinear velocity field, GARCH, height prediction

## Abstract

In this study, the basic concept of height nonlinear velocity field modeling in the CORS station is described. The noise results in a large deviation between the observation and predicted height. An ARCH testing method for heteroscedasticity of CORS height residual square series was proposed and the non-stationary characteristic of CORS height residual square time series was proved. A CORS height nonlinear velocity field reconstruction method based on the GARCH model was proposed. First, a nonlinear LS periodic fitting model was established for CORS height series data. Then, a GARCH model was established for the fitted non-stationary residual series. Finally, the signal term, linear trend term, and GARCH model noise term of nonlinear LS modeling were combined to reconstruct the nonlinear velocity field of the CORS height. The RMSE of nonlinear LS cycle modeling for 25 CORS stations worldwide ranged from 5 to 10 mm. The differences between the velocity, approximate annual and semi-annual amplitudes, and SOPAC results were 0.73 mm/a, 0.94 mm, and 0.51 mm, respectively. Compared with the centimeter amplitude of the CORS station height, the accuracy of the nonlinear model established in this study met the requirements. The results of height nonlinear velocity field reconstruction at 25 CORS stations worldwide showed that the mean square error of prediction of the one-year height movement reached 9 mm, and the average prediction accuracy of the semi-annual was 7 mm. Compared with the calculation accuracy of the current global CORS elevation component of 3–5 mm, the prediction error in this study was about 3 mm. The expected goal was achieved regarding the accuracy of the CORS station height nonlinear velocity field model.

## 1. Introduction

Continuously operating reference stations (CORS) coordinate motion prediction and analysis is an important basis for maintaining the accuracy and status of geocentric coordinate reference frame [1,2]. The research results show that the CORS station coordinate component sequence not only has the characteristics of linear change but also has the characteristics of nonlinear change. Apart from white noise, it also contains colored noise. Therefore, the noise term must be considered in the prediction model of the height nonlinear velocity field of the CORS station.

In terms of nonlinear motion analysis of CORS stations, there are obvious seasonal changes in the GPS station position time series [3]. Error analysis of the CORS station coordinate time series was conducted. The influence of colored noise on the velocity error of time series estimation was calculated, and the time series analysis software CATS was developed [4,5,6]. The effects of atmospheric pressure load and seven-parameter conversion on the geodetic estimation of geocentric motion and station elevation were studied [7], and noise in multivariable GPS position time series was analyzed [8]. Chinese experts have conducted a large number of studies: considering the establishment and maintenance method of the Earth reference frame considering nonlinear changes [9]; analyzing the motion characteristics of China’s CORS stations under CGCS2000 based on the nonlinear motion [10]; using the HHT technology to study the nonlinear time-variation of the time series of the base station height maintaining the CGCS2000 framework [11]; analyzing the phase law of the annual nonlinear variation of the coordinates of global GNSS stations [12]. Finally, the statistical correction model of the annual vertical variation of global GNSS stations was established [13].

In the aspect of velocity field modeling and noise model analysis of the CORS station, the noise of GPS coordinate time series was studied [14]. Based on the maximum likelihood estimation (MLE) criterion, different combinations of noise models are usually adopted to analyze the noise of the CORS station coordinate time series. Multi-year GPS observations were utilized to obtain velocity fields in Bulgaria and northern Greece [15], GNSS velocity field information was obtained through online PPP services [16], and the velocity field model was established using the data of continuously running GNSS stations [17]. The influence of snow depth, atmospheric pressure load, and soil moisture load on the displacement of the CORS station was calculated, and the noise characteristics of the CORS station coordinate sequence were mainly manifested as some combination noises [18]. The motion characteristics of the CGCS2000 framework and the singular spectrum nonlinear modeling technology of the nonlinear framework model are studied, which has obvious advantages compared with the modeling method of geophysical effect analysis [19]. The linear least squares model of the CORS station elevation time series data was established by using the method of given fixed period term, and then the solution was used as the iterative initial value of the nonlinear least squares model to realize the nonlinear modeling and prove the non-stationarity of the CORS station height motion [20].

In the study of the heteroscedastic characteristics of non-stationary time series, many models have been proposed, such as the conditional heteroscedastic [21], autoregressive conditional heteroscedastic [22], and threshold heteroscedastic [23]. The test of conditional heteroscedasticity in a time series was studied [24]. The GARCH model can be used to model and predict the stock market volatility of the Shanghai Composite Index [25]. The pseudo-likelihood method and its application to the functional GARCH model were discussed [26].

The CORS stations have accumulated continuous observations for more than 20 years, providing important information for the study of plate movement, geological disasters, etc. This study took the height time series data of global data from CORS stations of the Scripps Orbit and Permanent Array Center (SOPAC) from the past twenty years as the research object and established linear and nonlinear periodic fitting models, respectively. The GARCH (p, q) model was established for the non-stationary residual series after fitting. Finally, the nonlinear fitting model of elevation time series of CORS station and the GARCH model of non-stationary residual series were combined to obtain the nonlinear velocity field of elevation of CORS station, and the elevation motion was predicted.

## 2. Nonlinear Fitting of CORS Station Elevation

### 2.1. The Principle of Nonlinear Fitting of the Signal Term

The traditional linear least squares fitting model of the elevation coordinate component of the CORS station assigned fixed values to the periodic term [16], as shown in Equation (1):

(1) $$y=a+b\times t+A_{1}\sin(2\pi \times t)+A_{2}\sin(4\pi \times t)+A_{3}\sin(\pi \times t)$$

In Formula (1), a is the constant term, b is the linear rate, *A*_1_, *A*_2_, *A*_3_ are amplitude values corresponding to different period terms, and the corresponding fixed period values are $T_{1}=1,T_{2}=0.5,T_{3}=2$, respectively.

The nonlinear least squares fitting model takes each periodic term as the unknown value and adds the corresponding initial phase and frequency unknowns of each unknown periodic term, as shown in Equation (2):

(2) $$y=a+b\times t+A_{1}\sin(2\times \pi \times f_{1}\times t+\phi_{1})+A_{2}\sin(2\times \pi \times f_{2}\times t+\phi_{2})+A_{3}\sin(2\times \pi \times f_{3}\times t+\phi_{3})$$

In Formula (2): $f_{1}{,\;f}_{2}{,\;f}_{3}$ are the frequency values corresponding to different periodic terms, $\phi_{1},\phi_{2},\phi_{3}$ are the initial phase values corresponding to different periodic terms, and $a,\;b,\;t,\;A_{1},\;A_{2},\;A_{3},\;f_{1},\;f_{2},\;f_{3},\;\phi_{1},\;\phi_{2},\;\phi_{3}$ are the unknown parameters of the nonlinear least squares fitting model.

The idea of solving the unknown parameters of Equation (2) is as follows: the solution of the linear model (1) is used as the initial value of the nonlinear model (2), and the initial period values corresponding to the three main period items are: $T_{1}=1,T_{2}=0.5,T_{3}=2$. Expanding model (2) to the first-order term according to Taylor’s formula, the Gauss-Newton iterative algorithm is used to solve the unknown parameters. The iteration termination condition is set so that the difference between the values of the constant term a calculated in two adjacent calculations does not exceed 0.0001 m. The limit difference of iteration termination is one-tenth of the mm coordinate accuracy of the CORS station.

In order to evaluate the modeling and prediction effect of the CORS station, root mean squared error (RMSE) is used as the accuracy evaluation index, and RMSE reflects the deviation degree between the predicted value and the real value [20], in which n is the number of sample data, and Δd is the deviation.

(3) $$\mathrm{RMSE}=\sqrt{\sum {\left(\Delta d\right)}^{2}/n}$$

### 2.2. Nonlinear Fitting Experiment

**Figure 1:**
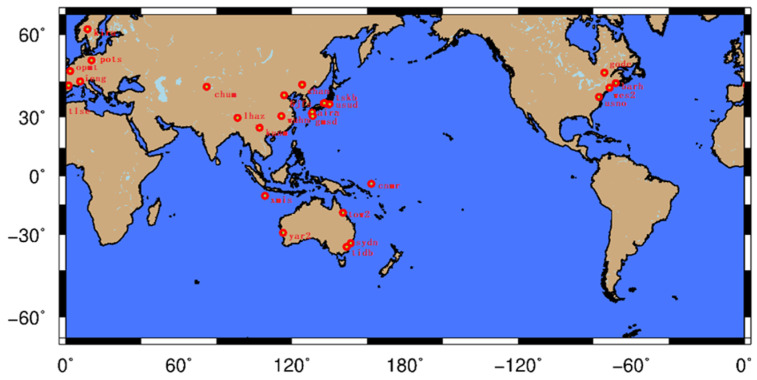
Distribution of 25 global CORS reference stations selected in the experiment.

The time series of elevation coordinates of 25 CORS stations from 2000 to 2020 were downloaded from the SOPAC global data center. The gross errors were removed from the data. For the missing points, cubic spline interpolation [21] was used, and the points were processed uniformly. The distribution is shown in Figure 1, and the points and roll call positions are shown in Table 1.

**Figure 2:**
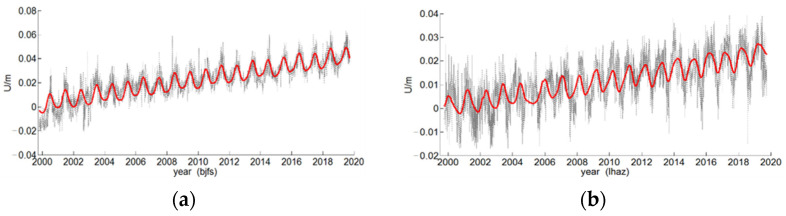
Fitting curve of bjfs (**a**) and lhaz (**b**) CORS height model.

Figure 2 shows the nonlinear modeling results of the elevation data of two CORS stations, bjfs and lhaz, in which the black dots represent the original elevation data values, and the red solid line represents the nonlinear modeling results. Table 2 shows the linearity of the two CORS stations. The parameter values of the model (1) and nonlinear model (2), the RMSE indicators of model (1) linear fitting and (2) nonlinear fitting are listed in Table 3, and the elevation data of the two CORS stations adopted nonlinear modeling. The RMSEs of both are smaller than those of the linear model, and the nonlinear modeling effect was better than that of the linear model.

**Figure 3:**
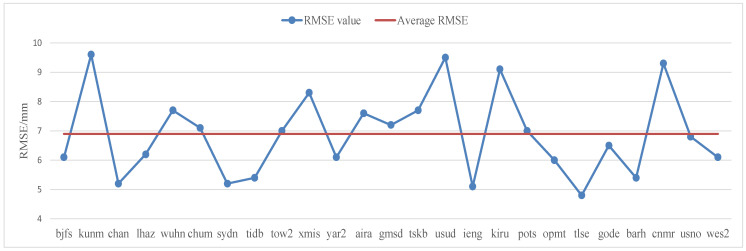
Distribution of RMSE indicators for 25 CORS elevation nonlinear LS period modeling around the world.

Table 4 shows the RMSE index of the nonlinear modeling of the elevation at 25 CORS stations around the world, and the RMSE distribution of the nonlinear modeling is shown in Figure 3. The average value of the RMSE index of nonlinear LS period modeling of elevation of 25 global base stations was 6.9 mm.

**Figure 4:**
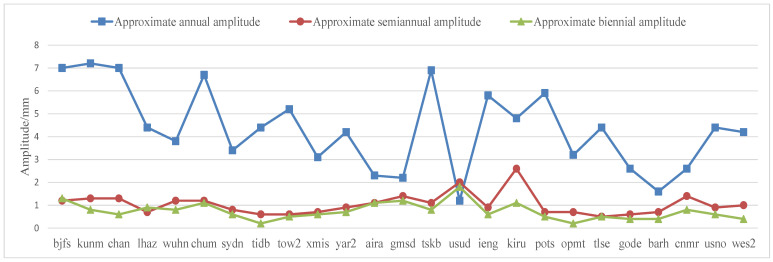
Amplitude value distribution (mm) of main periodic items of elevation nonlinear LS modeling of 25 CORS stations around the world.

The main period items of the elevation nonlinear modeling of 25 CORS stations around the world were counted, including the amplitudes of approximately one-year, half-year, and two-year periods, as shown in Table 5. In order to compare the amplitudes of the main period items more intuitively, three main periods were drawn. The distribution of the amplitude of the motion term is shown in Figure 4. The motion of the approximately one-year period was the main contribution term of the elevation motion of the CORS station. The one-year periodic amplitude of most CORS stations is 3–7 mm. The amplitude of the approximate half-year cycle term is 1~2 mm, and the approximate two-year cycle term was less than 1 mm. Except for the two stations of KIRU and USUD, the amplitudes of the approximately semi-annual and two-year cycles were about 2 mm, and the other 23 CORS stations were all less than 1.5 mm, and the amplitude of the approximately two-year cycle was generally smaller than that of the approximately semi-annual cycle.

The differences between the velocity of nonlinear LS period modeling, the amplitude of the approximately annual period, the amplitude of the approximate semi-annual period, and the SOPAC solution results are shown in Table 6. The median error of the difference between the two was calculated, the median error of the velocity field difference is 0.73 mm/a, the error of the annual amplitude difference is 0.94 mm, and the error of the half-year amplitude difference was 0.51 mm.

## 3. Non-Stationary Determination Method of Residual Squared Sequence

After establishing a nonlinear fitting model for the signal terms of the CORS station elevation motions, taking the sequence residuals as the research object, the ARCH test method and the residual squared sequence graph analysis method were used to judge the heteroscedasticity characteristics of the residual squared sequence and describe the variation of the residual squared sequence. 

### 3.1. ARCH Effect Test Method

**Figure 5:**
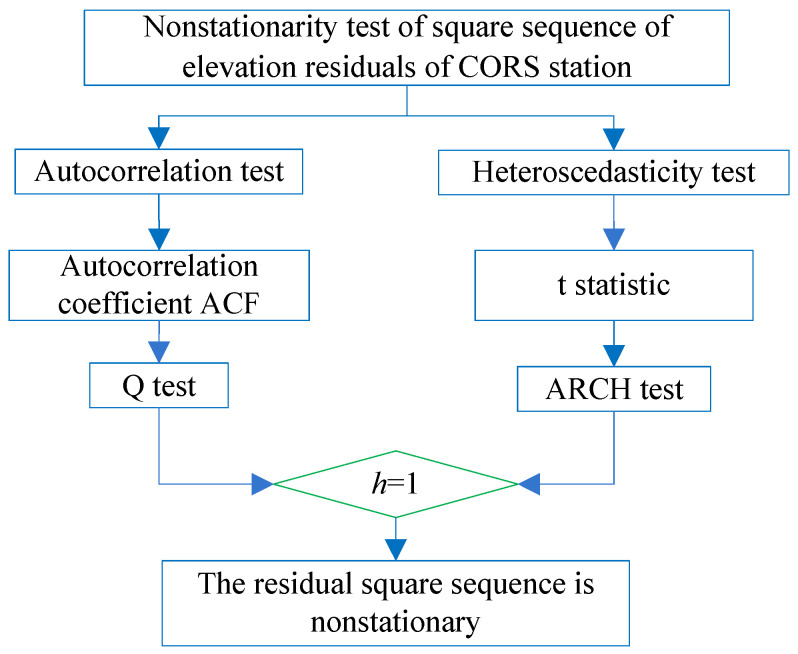
Non-stationarity test flow of CORS station elevation residual squared sequence.

**Figure 6:**
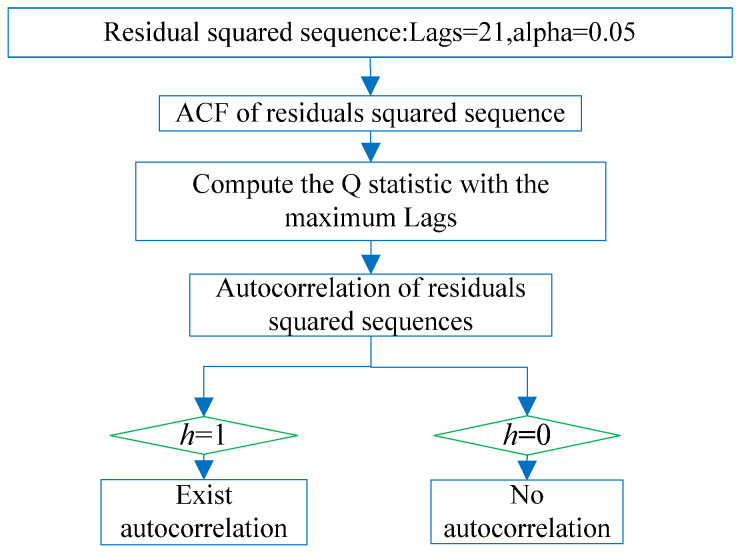
Autocorrelation Q test process.

**Figure 7:**
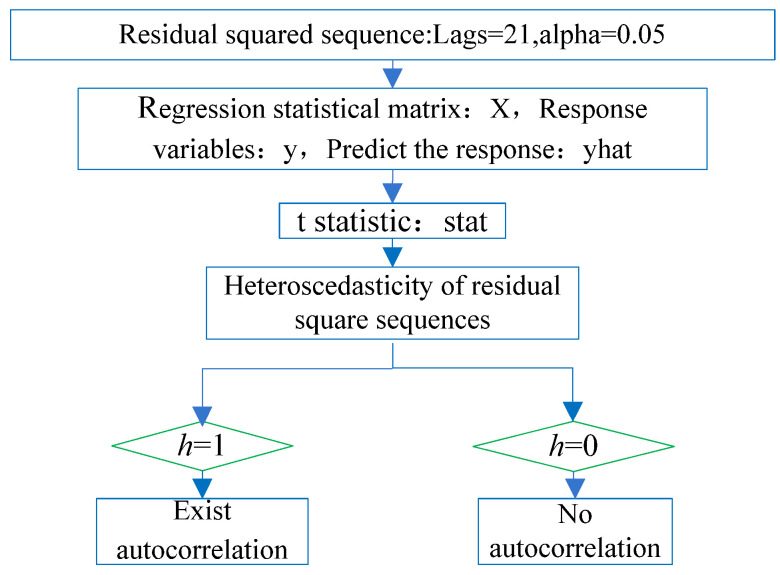
Heteroskedasticity ARCH test process.

The heteroscedasticity of the residual squared series meant that the value of a residual squared series greatly fluctuates over time. The residual squared series after nonlinear modeling of the CORS station elevation was tested to check whether the heteroscedasticity exists; that is, whether there is an ARCH effect in the residual squared series. The non-stationarity test is shown in Figure 5. It was divided into two steps. First, test the autocorrelation characteristics of the residual squared series using the autocorrelation coefficient (ACF) test of the residual squared series in Figure 6; second, test the heteroscedasticity of the residual squared series, and construct the t statistic in Figure 7. If there were both autocorrelation and heteroscedasticity in the residual squared series after nonlinear modeling of the CORS station elevation, it had an ARCH effect. That is, the residual squared series after nonlinear modeling of CORS station elevation was non-stationary.

If both autocorrelation and heteroscedasticity existed in the residual square sequence after elevation nonlinear modeling of CORS, then it had an ARCH effect. That is, the residual square sequence after elevation nonlinear modeling was non-stationarity.

The Q statistic for the autocorrelation test is defined as follows:

(4) $$Q_{LB}=T(T+2)\sum_{j=1}^{p}\frac{\rho_{j}^{2}}{T-j}$$

In the formula: $\rho_{j}$ is the *j*-order autocorrelation coefficient of the residual squared sequence, *T* is the total length of the residual squared sequence, and *p* is the set lag order.

The autocorrelation coefficient of the residual squared sequence is defined as follows:

(5) $$\mathrm{ACF}=r(s,t)/[{\left(DX(t)\cdot DX(s)\right)}^{0.5}]$$

In the formula, ACF represents the autocorrelation coefficient value of the residual squared, *r*(*s*,*t*) is the sequence autocovariance, and *DX*(*t*) and *DX*(*s*) represent the variance at different times. The sequence autocovariance and variance at different times are defined as:

(6) r(s,t)=E[(X(s)−E(X(s)))⋅(X(t)−E(X(t)))]

(7) $$DX(s)=E(X{\left(s\right)}^{2}-[E{\left(X(s)\right]}^{2},DX(t)=E(X{\left(t\right)}^{2}-[E{\left(X(t)\right]}^{2}$$

The partial autocorrelation coefficient (PACF) of the residual squared sequence is defined as follows,

(8) $$\phi_{kk}=\frac{D_{k}}{D}$$

$$D=\left|\begin{array}{cccc} 1 & \rho_{1} & \cdots & \rho_{k-1}\\ \rho_{1} & 1 & \cdots & \rho_{k-2}\\ \vdots & \vdots & \vdots & \vdots \\ \rho_{k-1} & \rho_{k-2} & \cdots & 1 \end{array}\right|\;D_{k}=\left|\begin{array}{cccc} 1 & \rho_{1} & \cdots & \rho_{1}\\ \rho_{1} & 1 & \cdots & \rho_{2}\\ \vdots & \vdots & \vdots & \vdots \\ \rho_{k-1} & \rho_{k-2} & \cdots & \rho_{k} \end{array}\right|$$

In the formula $\phi_{kk}$ represents the PACF with lag number *k*, which is obtained by Cramer’s rule, **D** is the coefficient determinant, $\rho_{i}$ is the autocorrelation coefficient of the sample, and **D***_k_* is the *k*th column of **D** replaced by a constant term.

During the heteroscedasticity *t*-test, the regression statistics matrix X was composed of the time corresponding to the residual squared sequence value, the response variable y is the residual squared sequence value, and the predicted response is the residual squared predicted value. The t statistic is defined as:

(9) $$t=\frac{\bar{X}-\mu }{\frac{\sigma_{x}}{\sqrt{n}}}$$

where $\bar{X}$ is the sample mean, μ is the mean of the overall residual squared sequence values, $\sigma_{x}$ is the sample standard deviation, and n is the sample size. In this study, the sample size was chosen to be 0.2 times that of the population.

### 3.2. Autocorrelation Test of Residual Squared Series

**Figure 8:**
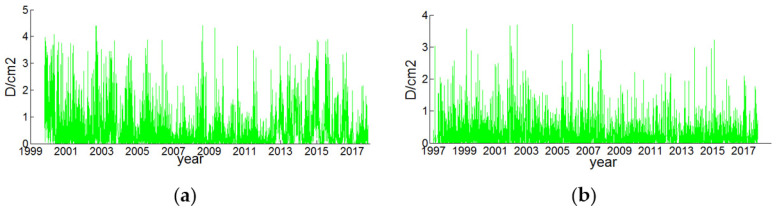
Residual squared sequence of elevations of two CORS stations, (**a**) bjfs and (**b**) bogo.

Autocorrelation means that the sequences are not completely independent of each other but have some kind of mutual relationship. The residual squared series of bjfs and bogo stations are shown in Figure 8. The vertical axis represents the residual squared value after nonlinear modeling of the elevation time series data, and the horizontal axis represents time.

**Figure 9:**
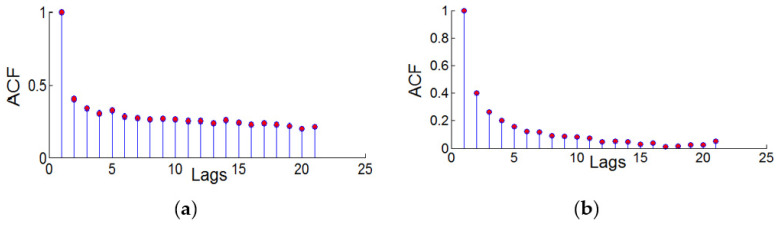
Autocorrelation plots of the squared series of elevation residuals of two CORS stations, (**a**) bjfs and (**b**) bogo.

In order to test the autocorrelation of the CORS elevation residual squared series, the autocorrelation of the residual squared series of the two CORS stations was drawn as shown in Figure 9. The coordinate ACF represents the autocorrelation coefficient of the reference station residual squared series, and the abscissa Lags represents the autocorrelation. The lag order value corresponds to the coefficient, the change of the autocorrelation coefficient ACF corresponds with the lag order. Lags do not rapidly approach 0, but slowly changes to 0; that is, the residual squared sequence has a certain relationship at different times, not independent of each other, the two-station residual squared sequence was qualitatively judged to have autocorrelation through the autocorrelation graph.

In order to quantitatively judge the autocorrelation of the squared residual series of the two stations, the Q-test method was used to test the autocorrelation of the squared residual series of the two stations. Lags = 21 were used to calculate the Q statistic, and the Q statistic of the bjfs and bogo stations were 4.12 and 4.23, respectively, which are both greater than the critical value of Q of 3.48 when the significance level is 0.05, so the null hypothesis was rejected (the residual squared sequence does not exist). The residual squared sequence of the two stations has antocorrelation(the logical quantity h = 1).

### 3.3. Heteroskedasticity Test of Residual Squared Sequence

The heteroskedasticity of the residual squared sequence was qualitatively judged from the residual squared sequence in Figure 8: the residual squared sequence of the two stations changed with time and were not a constant. There will be agglomeration every year or so (spikes and thick tails); that is, the residual squared sequence greatly fluctuated over time, and it was qualitatively judged that the residual squared sequence of the two stations had heteroscedasticity characteristics.

The ARCH test method was used to quantitatively determine the heteroscedasticity of the residual squared sequence. The *ARCH* process is as follows: *q* is the order of the ARCH process, *α*_0_ is a constant term, *α_i_* is the coefficient of the residual square value corresponding to the order of the ARCH process, vt is the random error, *α*_0_ > 0, *α_i_* ≥ 0 (*i* = 1, 2, …, *q*)

(10) $$\sigma_{t}^{2}=\alpha_{0}+\alpha_{1}\sigma_{t-1}^{2}+\cdots +\alpha_{i}\sigma_{t-i}^{2}+\upsilon_{t}$$

To calculate the *t* statistic stat of the residual squared series of the two stations: bjfs station stat = 2.98, bogo station stat = 2.81, both of which are greater than the t critical value 2.10 when the significance level is 0.05, so the null hypothesis is rejected (the residual squared series does not exist Heteroskedasticity), that is, the logical value h = 1, the residual squared sequence has heteroscedasticity, which proves that the two-station elevation residual squared sequence and the elevation residual sequence are non-stationary random processes.

## 4. GARCH Modeling of Non-Stationary Residual Series

The GARCH (*p*, *q*) model can effectively fit the heteroscedastic function with long-term memory, and the non-stationary time series data can be fitted and predicted by the GARCH model. The model is defined as Equation (11):

(11) $$\sigma_{t}^{2}=\alpha_{0}+\alpha_{1}\varepsilon_{t-1}^{2}+\cdots +\alpha_{j}\varepsilon_{t-j}^{2}+\beta_{1}\sigma_{t-1}^{2}+\cdots +\beta_{i}\sigma_{t-i}^{2}$$

In Formula (11), $\alpha_{0}$ is the constant term, $\alpha_{j}$ is the coefficient corresponding to the ARCH process, $\beta_{i}$ is the coefficient corresponding to the GARCH process, i=1,2,⋯p, j=1,2,⋯q, *p* is the model tailing value, and *q* is the model tailing value. The threshold of *p* was determined by the corresponding lag number when the residual series autocorrelation coefficient value ACF was basically stable, and the threshold value of *q* was determined by the corresponding lag number when the residual series partial autocorrelation coefficient value PACF was basically stable. The change of the autocorrelation coefficient with the number of lags was judged by the autocorrelation graph, and the change of the partial autocorrelation coefficient with the number of lags was judged by the partial autocorrelation graph.

To establish a GARCH (*p*, *q*) model for the residual series after the nonlinear fitting of the CORS station elevation, it was necessary to find the best (*p*, *q*) value. First, draw the autocorrelation diagram and partial autocorrelation diagram of the CORS station elevation motion residual sequence to obtain the threshold of (*p*, *q*), and then use the AIC criterion to determine the best (*p*, *q*) value.

**Figure 10:**
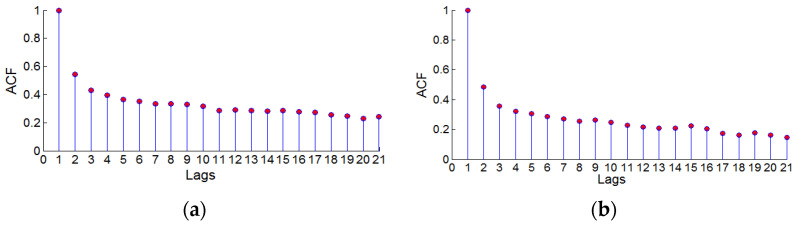
The autocorrelation graph of bjfs and lhaz CORS stations height residual sequence. (**a**) bjfs and (**b**) bogo.

**Figure 11:**
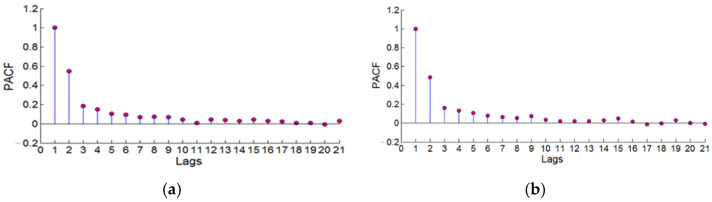
The partial autocorrelation graph of bjfs and lhaz CORS stations height residual sequence. (**a**) bjfs and (**b**) bogo.

Figure 10 and Figure 11 are the autocorrelation and partial autocorrelation diagrams of the two CORS station elevation residual sequences. The vertical axis ACF in Figure 10 represents the autocorrelation coefficient value of the residual sequence, and the horizontal axis lags represent the lag order corresponding to the autocorrelation coefficient value. The vertical axis PACF in Figure 11 represents the partial autocorrelation coefficient value of the residual sequence, and the horizontal axis lags represent the lag order corresponding to the partial autocorrelation coefficient value.

bjfs station: The autocorrelation coefficient value ACF tended to be stable after the lag order Lags = 11, and the partial autocorrelation coefficient value PACF tends to be stable after the lag order Lags = 7, that is, the threshold value of the tailing value *p* of the GARCH model is *p* ≤ 11. The threshold for the truncated value *q* was *q* ≤ 7.

lhaz station: The autocorrelation coefficient value ACF tended to be stable after the lag order Lags = 11, and the partial autocorrelation coefficient value PACF tended to be stable after the lag order Lags = 6; that is, the threshold value of the GARCH model trailing value *p* is *p* ≤ 11. The threshold for the truncated value *q* was *q* ≤ 6.

According to the AIC criterion, the optimal GARCH (*p*, *q*) model value of the bjfs station was (*p* = 1, *q* = 2), and the optimal GARCH (*p*, *q*) model of the lhaz station was (*p* = 1, *q* = 1).

The nonlinear model (2) corresponding to the epoch from 2000.001–2016.999 was used to fit the residual to establish the GARCH model. In order to evaluate the GARCH modeling effect, the residual error was predicted in the epoch from 2017.001–2018.999. GARCH (1, 2) and GARCH (1, 1) fitting models were established for the residual sequences of bjfs and lhaz, respectively. The parameter values of the GARCH model are shown in Table 7 and Table 8.

**Figure 12:**
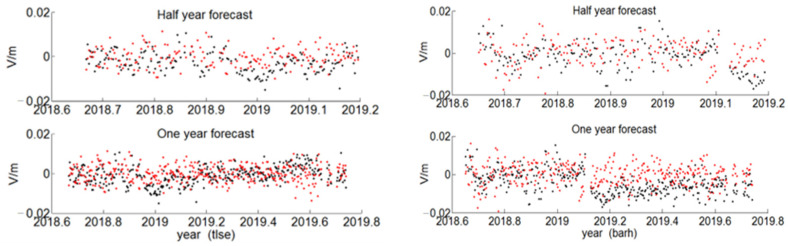
The GARCH residual sequence prediction of tlse and barh station.

**Figure 13:**
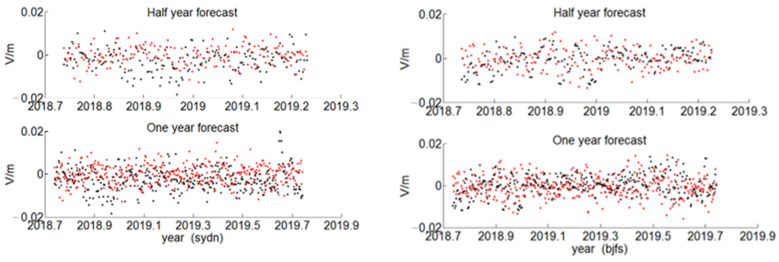
The GARCH residual sequence prediction of sydn and bjfs station.

Four representative stations, tlse, barh, sydn, and bjfs, were selected as the experimental data, and the residual sequence fitting results were obtained, as shown in Figure 12 and Figure 13. The black point represents the true value of the residual, which was obtained by fitting the nonlinear model (2) in the entire epoch interval, and the red point represents the predicted value of the residual GARCH model. It can be clearly seen from the two figures that the changes of the predicted residual value and the true residual value were basically the same.

In order to count the prediction effect of the residual GARCH model, the RMSE indicators of GARCH half-year and one-year forecasts are listed in Table 9. The GARCH forecasting accuracy of the residual series of the four stations is at the millimeter level.

## 5. Modeling of CORS Station Elevation Nonlinear Velocity Field

The idea of modeling the nonlinear velocity field of the CORS station elevation is as follows: Establish a nonlinear fitting model (2) for the signal term of the CORS station elevation motion, establish a GARCH model (3) for the residual sequence, and combine model (2) and model (3) to construct the elevation nonlinear velocity field of the CORS station. When predicting the nonlinear velocity field of CORS station elevation, a longer epoch interval was selected to establish the velocity field model, and a shorter epoch interval was used to predict the model accuracy.

### 5.1. Prediction Experiment of Elevation Velocity Field at Four CORS Stations

**Figure 14:**
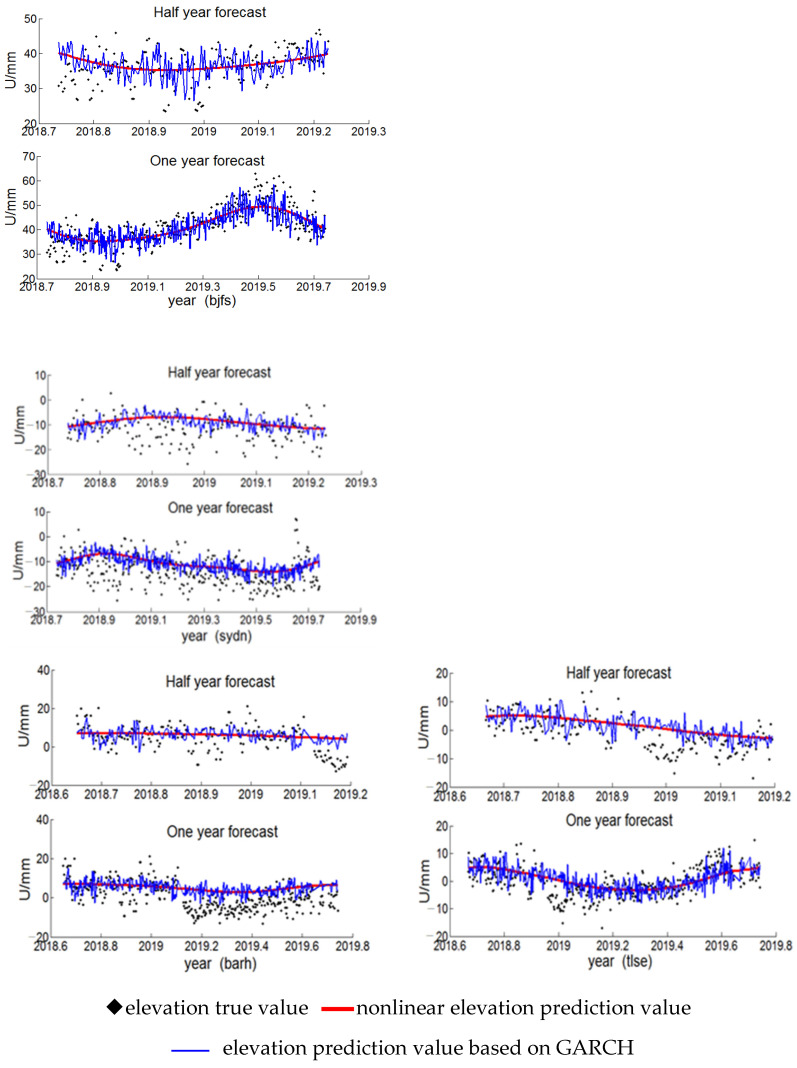
Prediction of four CORS station elevations based on GARCH nonlinear velocity field.

The nonlinear periodic fitting model (2) was established for the elevation data of the four CORS stations tlse, barh, sydn, and bjfs in the epoch from 2000.001–2016.999, and the GARCH model (3) was established by fitting the residuals. The elevation data of the epoch from 2017.001–2018.999 was used as a prediction sample. The effects of the two prediction methods for the CORS station elevation were compared and counted: one was the traditional nonlinear model (2), and the other was the prediction based on the combination of the GARCH residual model and the nonlinear periodic model, as shown in Figure 14, where the vertical axis represents the CORS station elevation value, the horizontal axis is the corresponding epoch, the black square represents the original elevation data, the red curve represents the prediction of the nonlinear model (2), and the blue line represents the prediction of the nonlinear model based on GARCH. Without considering the model fitting residuals, the fitting curve of the nonlinear model (2) was a smooth curve. After considering the noise term of the GARCH model, the prediction results were obviously closer to the real data. The traditional nonlinear prediction model lacked the noise term constraint, making the predicted elevation value greatly deviate from the trend of epoch change.

The model fitting residuals included the colored noise of the base station elevation motion and other approximate periodic terms that could not be fitted, which caused deviations between the modeled and actual values. In order to solve this problem, after fitting the above model, the colored noise (residual) was GARCH modeled, the coefficients of the GARCH (*p*, *q*) model were calculated, the CORS station elevation velocity field model was reconstructed based on the residual GARCH model and the nonlinear LS periodic motion term, and the reconstructed velocity was used. The field model performed elevation prediction (half- and one-year predictions) for the selected four CORS stations, analyzed the difference between the predicted value and the actual value, calculated the medium error of the prediction residual, and measured the accuracy of the reconstruction model of the elevation velocity field of the base station. As shown in Table 10, the accuracy of the nonlinear velocity field prediction model based on GRACH generally proved 1 mm better than the prediction accuracy of nonlinear LS alone.

### 5.2. Prediction of the Elevation Velocity Field of 25 CORS Stations around the World

**Figure 15:**
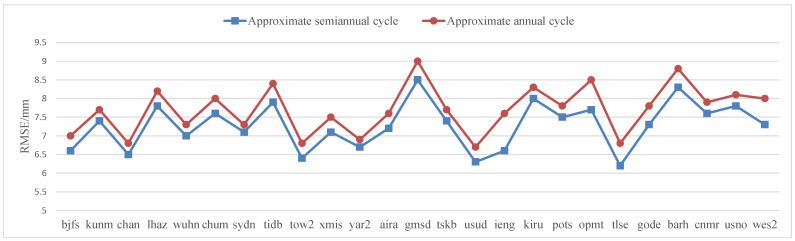
RMSE accuracy distribution of CORS station elevation nonlinear modeling residual prediction based on GARCH.

The accuracy of residual prediction of 25 CORS stations after nonlinear modeling of base station elevation based on GARCH model was counted, as shown in Table 11. The residual accuracy of approximate semi-annual prediction was generally higher than that of approximate annual period. Figure 15 is more intuitive. In Figure 15 the blue square polyline represents the RMSE accuracy distribution of approximate semiannual cycle, and the red dotted polyline represents the approximate annual cycle. The difference between the residual prediction accuracy of the annual and semi-annual approximate was given, and the general difference was about 0.5 mm.

**Figure 16:**
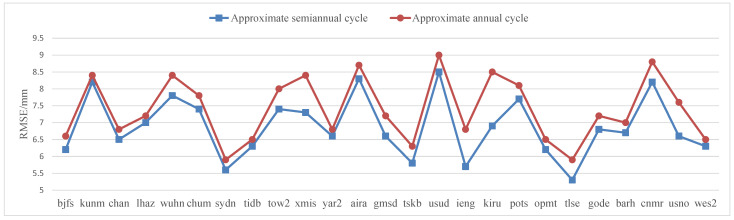
RMSE accuracy distribution of CORS station elevation nonlinear modeling residua based on GARCH+ nonlinear LS modeling.

The accuracy of the elevation predictions of 25 CORS stations after nonlinear modeling of base station elevation based on GARCH nonlinear LS modeling was counted, as shown in Table 12. The elevation accuracy of the approximate semi-annual prediction was generally higher than that of the approximate annual period. In Figure 16 the blue square polyline represents the RMSE accuracy distribution of approximate semiannual cycle, and the red dotted polyline represents the approximate annual cycle. It shows the difference in elevation prediction accuracy between approximate annual and semi-annual elevation prediction more intuitively, and the general difference was about 0.5 mm.

The nonlinear velocity field of the base station elevation combined with the GARCH residual and nonlinear LS periodic term models, the annual elevation prediction error was 9 mm, the semi-annual prediction accuracy was better than the annual prediction accuracy, and the semi-annual average prediction accuracy was at 7 mm. The highest accuracy of the point elevation obtained after the high-precision GNSS data processing of the station was at 3–5 mm, and the error between the elevation accuracy of the semi-annual prediction and the elevation accuracy of the base station was about 3 mm, which achieved the prediction effect.

## 6. Conclusions

1. We established a nonlinear fitting model for CORS station elevation time series data including approximate one-year, half-year, and two-year cycle terms. The annual period motion was the main contribution, and its amplitude was 3–7 mm, the half year and two-year were 1–2 mm and less than 1 mm, respectively. The median error of nonlinear fitting results of 25 CORS stations compared with SOPAC showed that the difference of velocity field was 0.73 mm/a, and the difference of annual period and half year period were 0.94 mm and 0.51 mm, respectively.
2. The GARCH fitting model of the CORS station elevation residual sequence was established, and the nonlinear velocity field of the CORS station elevation was obtained. After reconstructing the nonlinear velocity field of the elevation of the CORS station, the half-year prediction error was 7 mm. At present, the highest accuracy of the elevation coordinate component of the CORS station was 3–5 mm, and the difference between the two was about 3 mm.
3. The prediction results of different interval lengths showed that the prediction accuracy of the nonlinear velocity field model based on GARCH was better than the traditional nonlinear prediction model.

## Figures and Tables

**Table 1:** The names of 25 CORS stations around the world selected in the experiment.

Location	Station Name					
China and its surroundings	bjfs	kunm	chan	lhaz	wuhn	chum
Australia	sydn	tidb	tow2	xmis	yar2	
Japan	aira	gmsd	tskb	usud		
Europe	ieng	kiru	pots	opmt	tlse	
U.S.	gode	barh	cnmr	usno	wes2	

**Table 2:** Fitting parameters of bjfs and lhaz CORS height model.

Parameters	bjfs		lhaz	
	Linear	Nonlinear	Linear	Nonlinear
a/mm	2.60	2.70	0.44	0.32
b/mm	2.00	2.00	1.20	1.20
$A_{1}/\mathrm{mm}$	4.50	6.80	4.60	5.70
$A_{2}/\mathrm{mm}$	1.10	1.30	1.60	2.10
$A_{3}/\mathrm{mm}$	0.72	0.91	0.41	0.52
$f_{1}/a^{-1}$	1.0	1.01	2.0	1.01
$f_{2}/a^{-1}$	0.5	0.48	0.5	0.52
$f_{3}/a^{-1}$	2.0	2.08	2.0	2.09
$\phi_{1}/\mathrm{rad}$		1.85		0.48
$\phi_{2}/\mathrm{rad}$		6.79		0.29
$\phi_{3}/\mathrm{rad}$		4.50		4.78

**Table 3:** The RMSE index of bjfs and lhaz CORS height fitting.

Model	bjfs (mm)	lhaz (mm)
Linear model	7.9	6.0
Nonlinear model	6.0	5.4

**Table 4:** RMSE indicators of elevation nonlinear LS period modeling for 25 CORS stations around the world.

**Station Name**	**bjfs**	**kunm**	**chan**	**lhaz**	**wuhn**	**chum**	**sydn**	**tidb**	**tow2**
RMSE/mm	6.1	9.6	5.2	6.2	7.7	7.1	5.2	5.4	7.0
**Station Name**	**xmis**	**yar2**	**aira**	**gmsd**	**tskb**	**usud**	**ieng**	**kiru**	**pots**
RMSE/mm	8.3	6.1	7.6	7.2	7.7	9.5	5.1	9.1	7.0
**Station Name**	**opmt**	**tlse**	**gode**	**barh**	**cnmr**	**usno**	**wes2**		
RMSE/mm	6.0	4.8	6.5	5.4	9.3	6.8	6.1		

**Table 5:** Corresponding amplitude values (mm) of the main periodic terms of the elevation nonlinear LS modeling of 25 CORS stations around the world.

**Station Name/** **Amplitude**	**bjfs**	**kunm**	**chan**	**lhaz**	**wuhn**	**chum**	**sydn**	**tidb**	**tow2**
one year	7	7.2	7	4.4	3.8	6.7	3.4	4.4	5.2
half year	1.2	1.3	1.3	0.7	1.2	1.2	0.8	0.6	0.6
two years	1.3	0.8	0.6	0.9	0.8	1.1	0.6	0.2	0.5
**Station Name/** **Amplitude**	**xmis**	**yar2**	**aira**	**gmsd**	**tskb**	**usud**	**ieng**	**kiru**	**pots**
one year	3.1	4.2	2.3	2.2	6.9	1.2	5.8	4.8	5.9
half year	0.7	0.9	1.1	1.4	1.1	2	0.9	2.6	0.7
two years	0.6	0.7	1.1	1.2	0.8	1.8	0.6	1.1	0.5
**Station Name/** **Amplitude**	**opmt**	**tlse**	**gode**	**barh**	**cnmr**	**usno**	**wes2**		
one year	3.2	4.4	2.6	1.6	2.6	4.4	4.2		
half year	0.7	0.5	0.6	0.7	1.4	0.9	1		
two years	0.2	0.5	0.4	0.4	0.8	0.6	0.4		

**Table 6:** Comparison of elevation nonlinear LS period modeling and SOPAC results for 25 CORS stations around the world.

Station Name	Modeling Speed (mm/a)	Modeling Year Amplitude (mm)	Modeling Half Year Amplitude (mm)	SOPAC Speed (mm/a)	SOPAC Year Amplitude (mm)	SOPAC Half Year Amplitude (mm)	Speed Difference	Year Amplitude Difference	Half-Year Amplitude Difference
bjfs	2	7	1.2	2.43	6.97	0.95	−0.43	0.03	0.25
kunm	−0.9	7.2	1.3	1.31	8.12	0.33	−2.21	−0.92	0.97
chan	−0.4	7	1.3	−0.23	7.49	0.74	−0.17	−0.49	0.56
lhaz	1.2	4.4	0.7	1.17	6.02	1.63	0.03	−1.62	−0.93
wuhn	0.1	3.8	1.2	0.19	4.12	0.17	−0.09	−0.32	1.03
chum	0.4	6.7	1.2	0.4	7.72	1.83	0	−1.02	−0.63
sydn	−0.6	3.4	0.8	−0.75	3.54	0.26	0.15	−0.14	0.54
tidb	−1.1	4.4	0.6	−0.77	2.52	0.23	−0.33	1.88	0.37
tow2	−0.6	5.2	0.6	−0.64	2.9	0.96	0.04	2.3	−0.36
xmis	−0.1	3.1	0.7	−0.09	3.46	0.63	−0.01	−0.36	0.07
yar2	0.3	4.2	0.9	0.19	3.04	0.44	0.11	1.16	0.46
aira	−0.5	2.3	1.1	1.13	2.89	1.26	−1.63	−0.59	−0.16
gmsd	−0.8	2.2	1.4	0.56	2.18	0.74	−1.36	0.02	0.66
tskb	0.3	6.9	1.1	0.64	6.66	0.76	−0.34	0.24	0.34
usud	−0.5	1.2	2	−0.99	1.64	2.15	0.49	−0.44	−0.15
ieng	0.2	5.8	0.9	0.25	5.98	1.29	−0.05	−0.18	−0.39
kiru	6.8	4.8	2.6	6.76	5.02	2.28	0.04	−0.22	0.32
pots	0.3	5.9	0.7	0.42	5.86	0.27	−0.12	0.04	0.43
opmt	0.1	3.2	0.7	0.02	4.74	0.47	0.08	−1.54	0.23
tlse	0	4.4	0.5	0	4.33	0.63	0	0.07	−0.13
gode	−1.3	2.6	0.6	−1.2	3.1	0.89	−0.1	−0.5	−0.29
barh	0.3	1.6	0.7	0.25	2.53	1.04	0.05	−0.93	−0.34
cnmr	−2.3	2.6	1.4	−1.91	1.66	0.69	−0.39	0.94	0.71
usno	0.8	4.4	0.9	−0.78	3.87	0.79	1.58	0.53	0.11
wes2	0.2	4.2	1	0.27	3.38	1.05	−0.07	0.82	−0.05

**Table 7:** GARCH (1,2) model parameters of bjfs residual sequence.

Parameter	Value	Standard Error	t Statistic
Constant	0.0000010	0.0000003	4.0329500
GARCH(1)	0.9015880	0.0055081	163.68300
ARCH(2)	0.0703840	0.0048549	14.497700
Offset	0.0001350	0.0000712	1.8952600

**Table 8:** GARCH (1,1) model parameters of lhaz residual sequence.

Parameter	Value	Standard Error	t Statistic
Constant	0.0000117	0.0000059	19.896500
GARCH(1)	0.2877190	0.0297679	9.6654300
ARCH(1)	0.3148980	0.0230891	13.638400
Offset	0.0000204	0.0000673	0.0302618

**Table 9:** The GARCH residual sequence prediction RMSE of 4 CORS stations.

RMSE/mm	tlse	barh	sydn	bjfs
Half a year	5.3	6.7	5.6	6.2
A year	5.9	7.0	5.9	6.6

**Table 10:** Comparison of RMSE accuracy of elevation modeling predictions of four CORS stations (mm).

Method of Prediction	bjfs		sydn		barh		tlse	
	Half a Year	One Year	Half a Year	One Year	Half a Year	One Year	Half a Year	One Year
Nonlinear LS	7.2	7.5	6.4	6.8	7.4	7.7	6.0	6.8
nonlinear LS based GARCH	6.2	6.6	5.6	5.9	6.7	7.0	5.3	5.9

**Table 11:** RMSE statistics (mm) of residual prediction accuracy of CORS station elevation nonlinear modeling based on GARCH.

**Station Name/Section**	**bjfs**	**kunm**	**chan**	**lhaz**	**wuhn**	**chum**	**sydn**	**tidb**	**tow2**
half a year	6.6	7.4	6.5	7.8	7	7.6	7.1	7.9	6.4
one year	7	7.7	6.8	8.2	7.3	8	7.3	8.4	6.8
**Station Name/Section**	**xmis**	**yar2**	**aira**	**gmsd**	**tskb**	**usud**	**ieng**	**kiru**	**pots**
half a year	7.1	6.7	7.2	8.5	7.4	6.3	6.6	8	7.5
one year	7.5	6.9	7.6	9	7.7	6.7	7.6	8.3	7.8
**Station Name/Section**	**opmt**	**tlse**	**gode**	**barh**	**cnmr**	**usno**	**wes2**		
half a year	7.7	6.2	7.3	8.3	7.6	7.8	7.3		
one year	8.5	6.8	7.8	8.8	7.9	8.1	8		

**Table 12:** CORS station elevation prediction RMSE accuracy statistics based on GARCH+ nonlinear LS modeling.

**Station Name/** **Section**	**bjfs**	**kunm**	**chan**	**lhaz**	**wuhn**	**chum**	**sydn**	**tidb**	**tow2**
half a year	6.2	8.2	6.5	7	7.8	7.4	5.6	6.3	7.4
one year	6.6	8.4	6.8	7.2	8.4	7.8	5.9	6.5	8
**Station Name/** **Section**	**xmis**	**yar2**	**aira**	**gmsd**	**tskb**	**usud**	**ieng**	**kiru**	**pots**
half a year	7.3	6.6	8.3	6.6	5.8	8.5	5.7	6.9	7.7
one year	8.4	6.8	8.7	7.2	6.3	9	6.8	8.5	8.1
**Station Name/** **Section**	**opmt**	**tlse**	**gode**	**barh**	**cnmr**	**usno**	**wes2**		
half a year	6.2	5.3	6.8	6.7	8.2	6.6	6.3		
one year	6.5	5.9	7.2	7	8.8	7.6	6.5		

## Data Availability

Not applicable.
